# Oleispirillum naphthae gen. nov., sp. nov., a bacterium isolated from oil sludge, and proposal of Oleispirillaceae fam. nov.

**DOI:** 10.1099/ijsem.0.006292

**Published:** 2024-03-21

**Authors:** Chenghui Peng, Xue Zhang, Jiang Li, Min Yang, Shichun Ma, Hui Fan, Lirong Dai, Lei Cheng

**Affiliations:** 1 Key Laboratory of Development and Application of Rural Renewable Energy, Biogas Institute of Ministry of Agriculture and Rural Affairs, Chengdu 610041, Sichuan Province, PR China; 2 Center for Anaerobic Microbial Resources of Sichuan Province, Chengdu 610041, PR China; 3 National Agricultural Experimental Station for Microorganisms, Shuangliu, Chengdu 610213, Sichuan Province, PR China

**Keywords:** microaerophilic, nitrogen fixation, oil field, *Oleispirillaceae*, *Oleispirillum naphthae*

## Abstract

A microaerophilic, Gram-negative, motile, and spiral-shaped bacterium, designated Y-M2^T^, was isolated from oil sludge of Shengli oil field. The optimal growth condition of strain Y-M2^T^ was at 25 °C, pH 7.0, and in the absence of NaCl. The major polar lipid was phosphatidylethanolamine. The main cellular fatty acid was iso-C_17  :  0_ 3-OH. It contained Q-9 and Q-10 as the predominant quinones. The DNA G+C content was 68.1 mol%. Strain Y-M2^T^ showed the highest 16S rRNA gene sequence similarity to *Telmatospirillum siberiense* 26-4b^T^ (91.1 %). Phylogenetic analyses based on 16S rRNA gene and genomes showed that strain Y-M2^T^ formed a distinct cluster in the order *Rhodospirillales*. Genomic analysis showed that Y-M2^T^ possesses a complete nitrogen-fixation cluster which is phylogenetically close to that of methanogene. The *nif* cluster, encompassing the nitrogenase genes, was found in every N_2_-fixing strain within the order *Rhodospirillales*. Phylogeny, phenotype, chemotaxonomy, and genomic results demonstrated that strain Y-M2^T^ represents a novel species of a novel genus in a novel family *Oleispirillaceae* fam. nov. in the order *Rhodospirillales*, for which the name *Oleispirillum naphthae* gen. nov., sp. nov. was proposed. The type strain is Y-M2^T^ (=CCAM 827^T^=JCM 34765^T^).

## Introduction

Members of the order *Rhodospirillales*, affiliated with the class *Alphaproteobacteria* within the phylum *Pseudomonadota*, are ubiquitous in soil [1,2], air [3], water [4,5], anode biofilms [6], crude oil-contaminated soil [7,8], respiratory secretions [9], and other niches [10,13]. The order *Rhodospirillales* comprises 12 validly published families which were reclassified by Hördt *et al.* in 2020 [14]. However, six more novel families were separated from the existing families within this order according to the phylogenomic analyses [15]. The family *Rhodospirillaceae* was split into family ‘*Dongiaceae*’ (including genera *Dongia*, *Aliidongia*, and *Hypericibacter*), ‘*Oceanibaculaceae*’ (*Oceanibaculum*), and current *Rhodospirillaceae* (*Pararhodospirillum*, *Phaeovibrio*, *Rhodospira*, *Rhodospirillum* and *Roseospirillum*) [15]. Most members of this order are chemoorganoheterotrophic and/or photoorganoheterotrophic [16,18]. It also contains free-living nitrogen-fixing bacteria, such as the genus *Azospirillum* in the family *Azospirillaceae* [13,19, 20]. Most of them were obtained from plant root-related environments, including the facultative anaerobes *Azospirillum oryzae* COC8^T^ [19] and *Azospirillum lipoferum* Sp 7^T^ [6,21]. There are several nitrogen-fixing bacteria within *Azospirillum* (*Azospirillum palustre* B2^T^, *Azospirillum oleiclasticum* RWY-5-1-1^T^ and *Azospirillum rugosum* IMMIB AFH-6^T^) and aerobic *Oleiliquidispirillum nitrogeniifigens* 64-1^T^ in the family *Rhodospirillaceae* isolated from oil-related environments, including oil-contaminated soil and oil production mixtures [7,8, 13], indicating that members of *Rhodospirillales* are probably involved nitrogen cycling in oilfield.

Biological nitrogen fixation, in which micro-organisms convert atmospheric nitrogen gas (N_2_) to ammonia (NH_3_), is an important mechanism to support bio-available nitrogen for organisms. Additionally, it plays a critical role in the global nitrogen cycle. It has been shown that this process is mainly mediated by three different nitrogenases, molybdenum–iron nitrogenase (Nif, Mo-Fe), vanadium–iron nitrogenase (Vnf, V-Fe), and iron-only nitrogenase (Anf, Fe-Fe) [22,23]. Nif has two protein components: an electron delivery component (NifH, encoded by *nifH*) and a catalytic component (NifD and NifK, encoded by *nifD* and *nifK*, respectively) [23]. Nif is the most important nitrogen-fixation protein and is widely distributed in archaea (phylum *Euryarchaeota*) and 13 phyla in the bacterial domain, primarily including *Bacillota*, *Bacteroidota*, *Cyanobacteria* and *Pseudomonadota* [23], which have been found in a variety of environments ranging from marine [24,25], rhizosphere [26,27], freshwater sediments [24], and oilfields [8,28]. Genomic studies have revealed that nitrogen fixation-related genes constitute a nitrogen fixation cluster, such as the 17 *nif* genes (*nifQ*, *nifA*, *nifL*, *nifH*, *nifD*, *nifK*, *nifT*, *nifY*, *nifE*, *nifN*, *nifX*, *nifS*, *nifU*, *nifW*, *nifZ*, *nifM*, and *nifF*) distributed in a 49 kb region in *Stutzerimonas stutzeri* A1501^T^ [29,30]. Previous studies have demonstrated that all nitrogen fixation bacteria in the order *Rhodospirillales* carry *nif* gene clusters, and no *vnf* or *anf* cluster has been identified in these species [6,8,13, 19, 28, 31].

## Isolation

Strain Y-M2^T^ was isolated from oil sludge collected from Shengli oil field in Shandong province, PR China (38° 39′ 28″ N 104° 04′ 59″ E) using a traditional dilution method with a 96-well microplate as described previously [32]. Mineral medium containing 9.0 g NaCl, 0.5 g KCl, 0.3 g NH_4_Cl, 0.2 g KH_2_PO_4_, 3.0 g MgCl_2_·6H_2_O, 0.15 g CaCl_2_·2H_2_O, 0.5 g l-cysteine hydrochloride, 2 ml TE284 buffer, and 0.001 g resazurin in 1000 ml distilled water was used for isolation. The medium was boiled and cooled under 99.999 % N_2_ flow, dispensed into vials or tubes with butyl rubber stoppers, then sterilized at 121 °C for 30 min. The initial culture (0.5 ml) was diluted 10-fold by adding into 4.5 ml medium supplemented with short-chain fatty acids, glucose, yeast extract, and tryptone. After 7–30 days of incubation at 25 °C in rectangular jar using AnaeroGen (Thermo Scientific), pure cultures were isolated and transferred into fresh liquid medium. The purified strain Y-M2^T^ was cultured at 25 °C under microaerobic conditions (10 % oxygen) in modified Reasoner’s 2A (R2A) broth containing 0.25 g tryptone, 0.5 g casein hydrolysate, 0.5 g yeast extract, 0.5 g soluble starch, 0.3 g K_2_HPO_4_, 0.1 g MgSO_4_, 0.3 g sodium pyruvate, 0.25 g peptone, 0.5 g glucose, and 1000 ml distilled water. The medium was prepared and dispensed under N_2_ with the addition of 10 % oxygen, and sterilized at 121 °C for 15 min.

## Genome sequencing

Strain Y-M2^T^ grew in 300 ml modified R2A medium at 28 °C for 48 h and 180 r.p.m. was harvested for genomic DNA sequencing. A commercial bacterial genomic DNA extraction kit (Magen) was used to extract the genomic DNA of strain Y-M2^T^. The draft genome of strain Y-M2^T^ was sequenced and assembled using the Illumina NovaSeq sequencing platform (Beijing Novogene Technology Co., Ltd., Beijing, PR China). The SPAdes strategy was used to assemble the genome, and the resulting N20, N50, and N90 values. Genome quality and single-copy marker genes were retrieved using CheckM (version 1.1.0; (https://github.com/Ecogenomics/CheckM/wiki).

## Genome features

The genome shotgun project of strain Y-M2^T^ has been deposited at DDBJ/ENA/GenBank under the accession JAQAZG000000000. The N20, N50, and N90 values of strain Y-M2^T^ were 461 460, 216 590, and 128 336, respectively. The draft genome sequence of strain Y-M2^T^ was 3 214 613 bp, contained 2903 open reading frames, 27 contigs, three rRNAs (one each of 5S rRNA, 16S rRNA, and 23S rRNA), 48 tRNA, and 69 ncRNA. The DNA G+C content was 68.1 mol % (Table S1, available in the online version of this article).

## Phylogeny

The 16S rRNA gene sequence was retrieved from the genome (GenBank accession number MZ270535.1) and compared with the sequence obtained by amplifying with the universal bacterial primers 27F (5′-AGAGTTTGATCMTGGCTCAG-3′) and 1492R (5′- TACGGYTACCTTGTTACGACTT-3′) [33] to confirm the purity of strain Y-M2^T^. The 16S rRNA gene sequences of strains with a close taxonomic relationship were downloaded from the NCBI (www.ncbi.nlm.nih.gov) and LPSN databases (http://www.bacterio.net/). All sequences were aligned using muscle (version 3.8.1551) [34], and the phylogenetic trees were reconstructed using the software FastTree (version 2.1.11) with default parameters [35], and iq-tree (version 2.0.3) with ‘-m TEST -bb 1000 -alrt 1000’ [36].

**Fig. 1.**
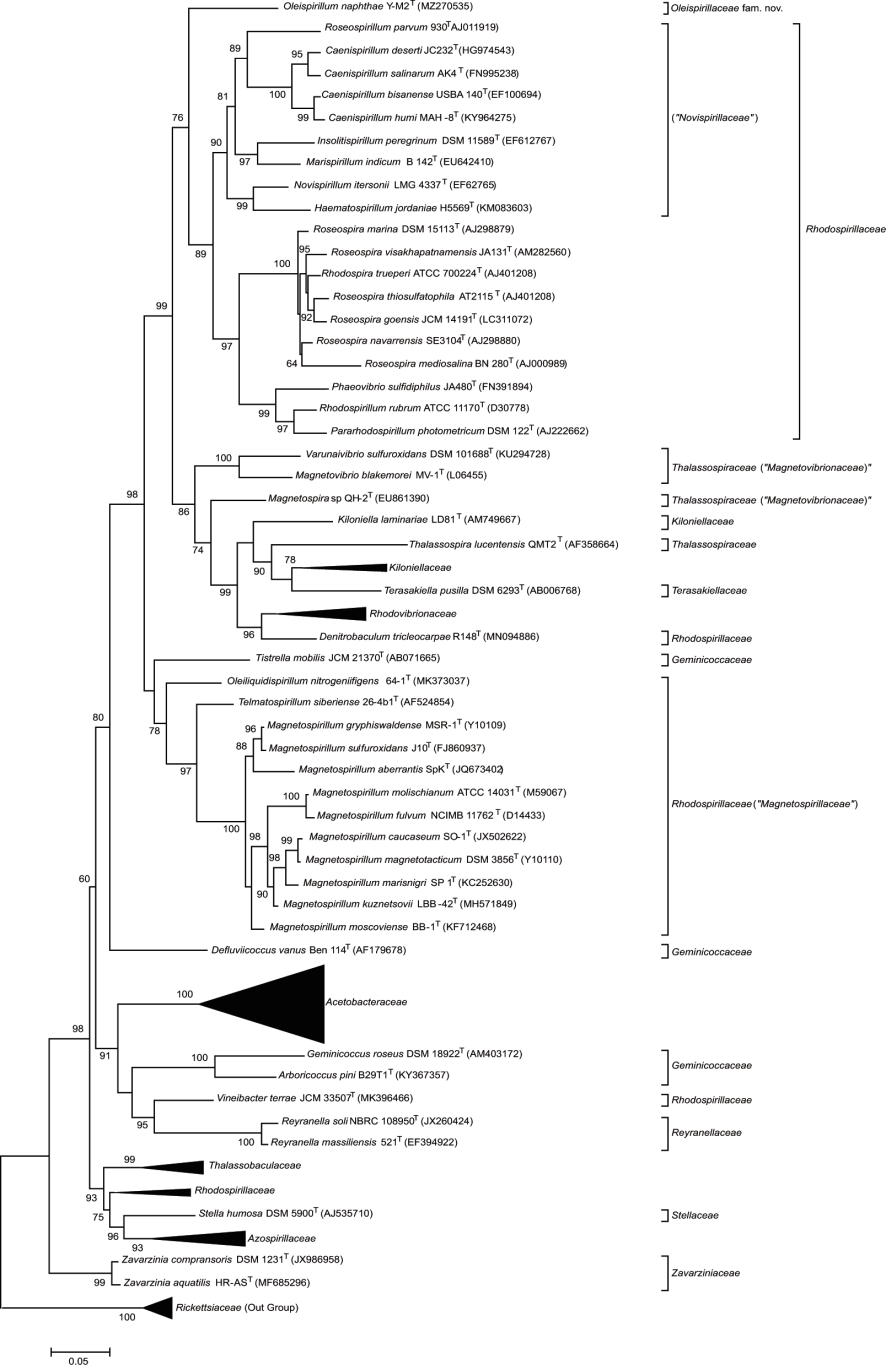
Phylogenetic trees of strain Y-M2^T^ and *Rhodospirillales* members based on 16S rRNA genes. Species of the genus *Rickettsia* within the order *Rickettsiales* were used as an outgroup. Bootstrap percentages are based on 1000 replications.

**Fig. 2.**
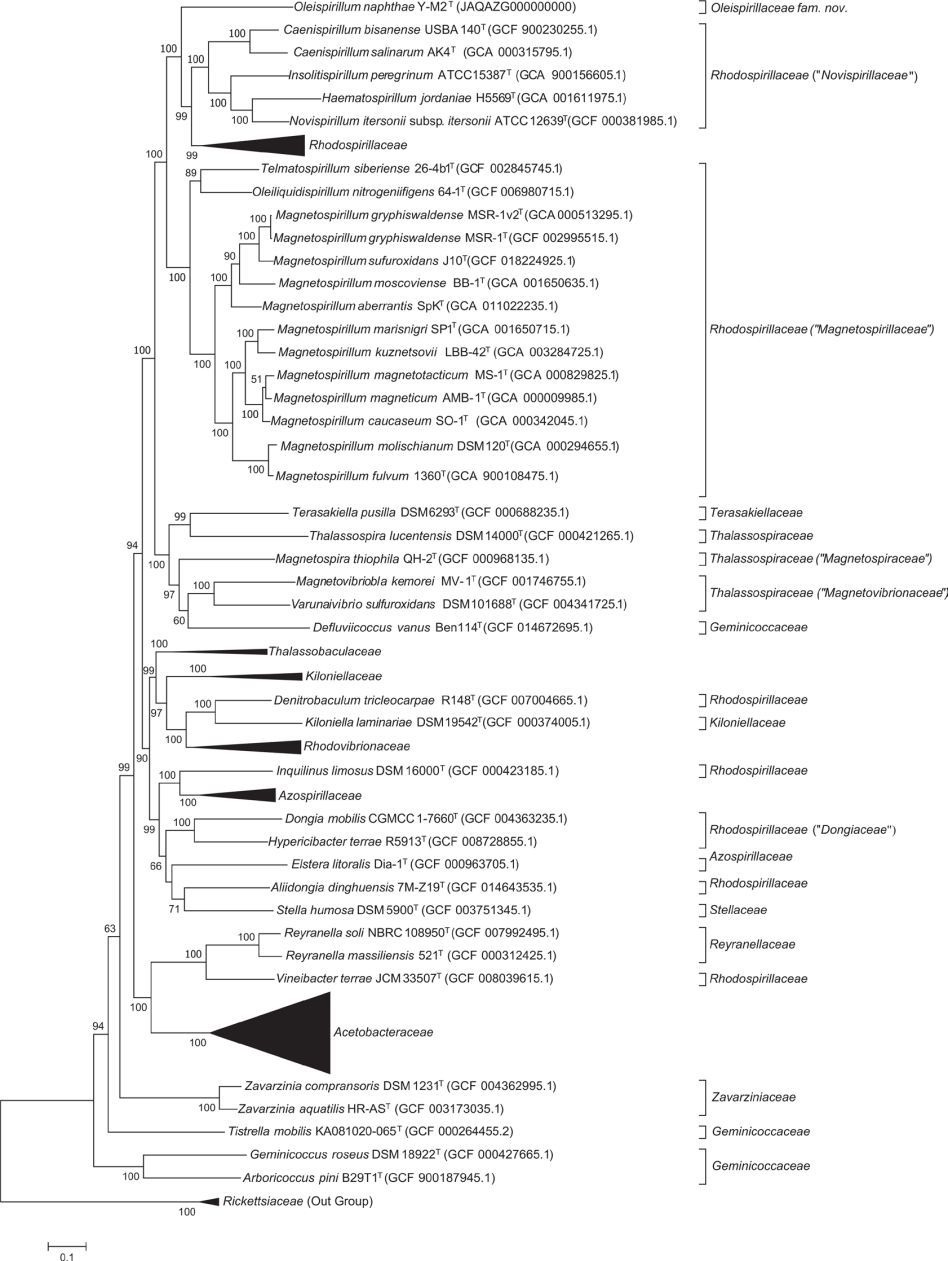
Phylogenomic analyses of *Rhodospirillales* members based on 120 marker genes reconstructed by GTDB-tk.

The 16S rRNA gene sequence of strain Y-M2^T^ containing 1487 bp nucleotides was used for phylogenetic analyses, and the gene sequence similarity revealed that strain Y-M2^T^ was most closely related to *T. siberiense* 26-4b1^T^ (91.1 %) and *Magnetospirillum gryphiswaldense* MSR-1^T^ (90.6 %) in the family ‘*Magnetospirillaceae*’ (formerly belonging to *Rhodospirillaceae*) of the order *Rhodospirillales*. Based on the thresholds of sequence identity of 16S rRNA genes for separating new genera (94.5 %) [37], strain Y-M2^T^ is proposed to represent a novel genus in order *Rhodospirillales*. The maximum-likelihood phylogenetic tree based on 16S rRNA gene sequences showed that strain Y-M2^T^ located in the clade containing species of the family ‘*Novispirillaceae*’ and *Rhodospirillaceae*, but formed an independent branch which was well separated from all published families of the order *Rhodospirillales* (Fig. 1). The separation of strain Y-M2 ^T^ from all other valid families also occurred in the neighboir-joining and minimum-evolution phylogenetic trees (Fig. S1A and B). The average 16S rRNA gene sequence similarities from phylogenetic analysis between strain Y-M2^T^ and species within ‘*Magnetospirillaceae*’, ‘*Novispirillaceae*’, and *Rhodospirillaceae* were ≤90.8 % (Table S2), which was close to the minimum sequence identity for distinguishing families (87.7 %). Phylogenomic trees reconstructed using GTDB-tk (Fig. 2) and using concatenated alignment of 92 core genes (Fig. S1C) further confirmed that strain Y-M2^T^ is independent of ‘*Magnetospirillaceae*’, ‘*Novispirillaceae*’, *Rhodospirillaceae*, as well as other *Rhodospirillales* families, indicating it may represent a new family in order *Rhodospirillales*.

Paired genomic average nucleotide identity (ANI) and average amino acid identity (AAI) were calculated using OrthoANIu [38] and Compare M (https://github.com/dparks1134/CompareM), respectively. Percentages of conserved proteins (POCP) between two microbial genomes were calculated according to the method described previously [39]. The AAI values between strain Y-M2^T^ and the genera of ‘*Magnetospirillaceae*’, ‘*Novispirillaceae*’, and *Rhodospirillaceae* were ≤60.2 % (Table S2), close to the family delineating threshold (60.0 %) of the order *Rhodospirillales* [15], supporting the proposal of a novel family. ANI and POCP values were less than 71.8 and 48.0 %, respectively (Table S2).

## Comparative genomics

For genomic comparison analyses, a total of 447 genomes belonging to the order *Rhodospirillales* and 23 genomes of type strains belong to the order *Rickettsiales* in class *Alphaproteobacteria* were downloaded from the NCBI database. These genomes were predicted by Prodigal (version 2.6.3) [40] and annotated by using KofamKOALA with default parameters [41].

**Fig. 3.**
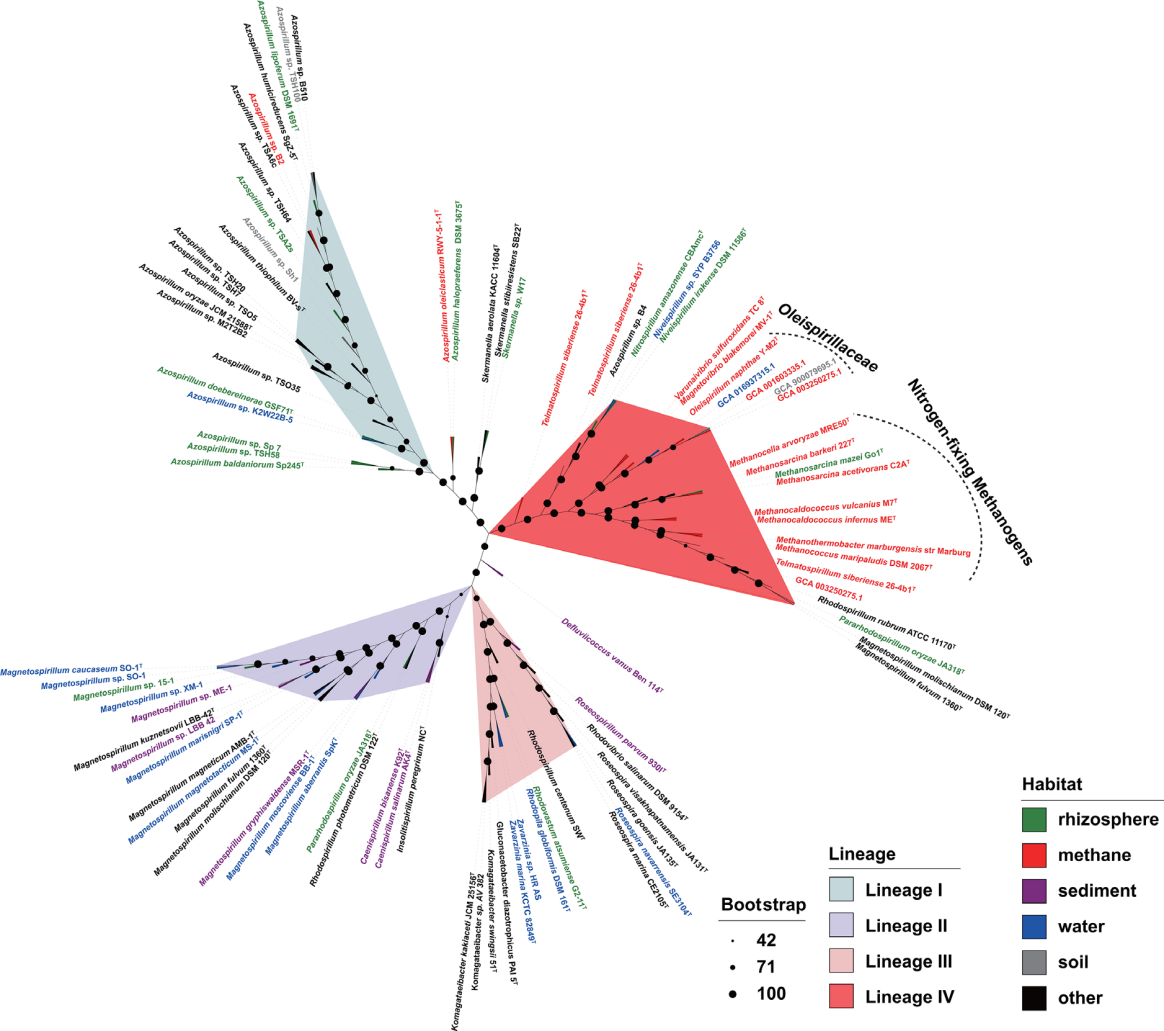
Maximum-likelihood tree reconstructed by concatenation of *nifHDK* genes. This tree includes the genomes of 45 nitrogen-fixing *Rhodospirillales* species and eight archaea nitrogen-fixing species from a variety of environments. Nodes with bootstrap values are marked with different size black dots. The colours of the labels and clades indicate the habitat where the strain was isolated and different lineages of *nifHDK* concatenation, respectively.

Strain Y-M2^T^ contains the *nif* family genes *nifH*, *nifD*, and *nifK*, which are key genes related to nitrogen fixation. *nifH* encodes Fe protein, which is a homodimer bridged by an inter-subunit (4Fe-4S) cluster that serves as the obligate electron donor for the MoFe protein, *nifDK* encodes Mo-nitrogenase, which is composed of dinitrogenase (MoFe protein) and dinitrogenase reductase (Fe protein) [42]. *nifH, nifD*, and *nifK* act as marker genes for predicting the nitrogen fixation capabilities of microbes [43,45]. Therefore, strain Y-M2^T^ was speculated to have the ability to fix atmospheric nitrogen. To elucidate the adaptive mechanisms of the *nif* gene of Y-M2^T^, a comparative genomic analysis of the chromosomal regions flanking the *nif* gene clusters contained within a contig. The *nif* cluster genes *nifH*, *nifD*, and *nifK* were present in all N_2_-fixing strains in the order *Rhodospirillales*, whereas partial genes of the *anf* and *vnf* clusters were only found in *M. fulvum* DSM 113^T^, *M. molischianum* DSM120^T^, *Rhodospirillum rubrum* ATCC 11170^T^, and *T. siberiense* 26-4b^T^. Further analysis revealed that the *anfG* gene mediates crosstalk between the gene *nifH* and *nifK* encoding nitrogenases components [46], for which the assignments were similar to the archaea *Methanosarcina activorans* C2A^T^ (Fig. 3).

The maximum-likelihood tree was reconstructed by concatenation sequence of *nifHDK* genes, and the type of *nifHKD* was distincted to four distinct lineages (Fig. 3). Lineages I, II and III were most related to the genera *Azospirillum*, *Magnetospirillum*, and *Roseospirillum*. Interestingly, strain Y-M2^T^, as well as several species of the family *Thalassospiraceae* and *Rhodospirillaceae*, were placed in the lineage IV, which also contains archaea belonging to the genera *Methanosarcina*, *Methanocella*, *Methanocaldococcus*, *Methanococcus*, and *Methanothermobacter*, implying the homology of their nitrogenases (Fig. 3). In addition, most of bacteria and archaea in lineage IV were isolated from anaerobic or methanogenic environments (Fig. 3). A previous study has revealed that the distribution of nitrogenase genes was crucially impacted by horizontal gene transfer (HGT) events, the acquisition of *nif* by *Firmicutes* possibly through HGT events with a methanogen ancestor [47]. Therefore, the phylogenetic relationships among strain Y-M2^T^, *Thalassospiraceae*, *Rhodospirillaceae* species, and archaea in the *nifHDK* phylogenetic tree suggested the HGT events possibly occurred between *Rhodospirillales* and methanogens in anoxic environments (Fig. 3).

To further analyse *nifHDK* lineage IV, we reconstructed the *nif* gene organization based on encoding genes sequences. The *nif* clusters in the lineage IV could be classified to three distinct types (Fig. S2). The type I *nif* gene cluster was the most common nitrogenase in bacteria. Compared to type I, type II had an additional *anfG* gene inserted between *nifH* and *nifK*, whereas type III had genes *nifN* and *nifE* which replaced *nifT* in type I (Fig. S2). Surprisingly, the organization of *nif* genes showed that * T. siberiense* 26-4b1^T^ contained all types of *nif* cluster described above, which indicates that it may play an important role in the evolution of the *nif* genes in the order *Rhodospirillales*.

## Morphology, physiology and chemotaxonomy

For morphology tests, the strain Y-M2^T^ was incubated at 25 °C in R2A medium for 7 days. Cell size, shape, and flagella were assessed using a scanning electron microscope (jeol JEM-1400 Plus) and a transmission electron microscope (jeol JEM-1230). Gram-stain and spore-stain kits (Solarbio) were used to assess Gram staining and spore formation, respectively. Growth of strain Y-M2^T^ at different temperatures (15, 20, 25, 30, and 37 °C), pH values (pH 5.5–8.0 at intervals of 0.5 pH units), and NaCl concentrations (0–70 g l^−1^ at intervals of 10 g l^−1^ NaCl) was determined in R2A broth by measuring the OD_600_ value with a spectrometer (DU730, Beckman Coulter). The following sterilized anoxic buffers at a final concentration of 20 mM were added into the fresh medium: MES (pH 5.5, 6.0, 6.5), PIPES (pH 7.0, 7.5), Tris (pH 8.0). The pH was finally adjusted by addition of solutions of HCl or NaOH before incubation, which was determined with a pH meter (Horiba). Oxygen requirement was tested under anaerobic, 2 %, 10% and 20 % of O_2_ (v/v) following the procedure described previously [48]. Biochemical characteristics of strain Y-M2^T^ were determined using API 20NE, API ZYM and API 20E kits (bioMérieux) according to the manufacturer’s instructions. Microbial nitrogenase (NITS) ELISA scientific research kit (Jingmei) was used to determine the content of nitrogenase. All experiments mentioned above were performed in triplicates. Cultures of strain Y-M2^T^ and *T. siberiense* 26-4b^T^ incubated at 25 °C in R2A medium were harvested at logarithmic phase for fatty acids analyses. The protocols of Microbial Identification Inc. (midi) and gas chromatography (Agilent 6990) were used to identify fatty acids using Sherlock software (version 6.3) [49]. Respiratory quinones were detected in strain Y-M2^T^ using a previously described protocol [50,51]. Polar lipids were extracted using a chloroform–methanol system and were analysed using one- and two-dimensional thin-layer chromatography following the method described by Kates *et al*. [52].

Cells of strain Y-M2^T^ were Gram-negative, spiral-shaped with motility with monotrichous flagella, 0.8–3.0 µm long, 0.2–0.4 µm wide (Fig. S3), and non-spore-forming. Growth was observed at 20–30 °C, pH 6.0–7.5, without NaCl, and at 2–10 % O_2_. Optimal growth occurred at 25 °C, pH 7.0, without NaCl, and in the presence of 10 % O_2_.

Positive for acetoin (acetyl methylcarbinol) produced by glucose fermentation via the butylene glycol metabolic pathway and the production of the enzyme gelatinase which liquefies gelatin in API 20E test. Positive for arginine dihydrolase, urease, hydrolysis aesculin and β-galactosidase, negative for reduction of nitrates to nitrites or nitrogen in API 20NE tests (Table 1). In API ZYM tests, strain Y-M2^T^ presented positive reactions for alkaline phosphatase, esterase (C4), lipid esterase (C8), leucine arylaminase, acid phosphatase, naphthol AS-BI phosphate hydrolase, β-galactosidase, and β-glucosidase, and negative reactions for lipoid enzyme (C14), valine arylaminase, cystine arylaminase, trypsin, chymotrypsin, α-galactosidase, uronic acid glycosidase, α-glucosidase, *N*-acetylglucosamine enzyme, α-mannosidase, nd β-fucosidase. In addition, 335.7 ng l^−1^ nitrogenase was detected in about 0.1 ml of biomass of strain Y-M2^T^ (Fig. S4), indicating nitrogenase is positive in strain Y-M2^T^. Strain Y-M2^T^ contained Q-9 and Q-10 as the predominant quinones (14.2 and 85.2  %, respectively). The major fatty acids were iso C_17 : 0_ 3-OH (26.1 %), summed feature 8 (15.3 %), summed feature 3 (13.3 %), and iso-C_15 : 0_ 3-OH (12.6 %), which were different from the closest related strain containing C_18 : 1_* ω*7*c*, C_16 : 0_, and C_17 : 0_ as the major fatty acids (Table 1). The polar lipid profile comprised phosphatidylethanolamine, aminolipid, and aminophospholipidase as the main polar lipids (Fig. S5).

**Table 1.** Differential phenotypic characteristics of Y-M2^T^ compared with representative species of each family within the order *Rhodospirillales* Strains: 1, Y-M2^T^ (this study); 2, *T. siberiense* 26-4b^T^ [28]; 3, *M. gryphiswaldense* MSR-1^T^ [31,53]; 4, *A. aceti* NBRC 14818^T^ [53]; 5, *A. lipoferum* DSM 1691^T^ [20]; 6, *G. roseus* D2-3^T^ [54]; 7, *K. laminariae* LD81^T^ [55]; 8, *R. massiliensis* 521^T^ [53]; 9, *R. salinarum* DSM 9154^T^ [53]; 10, *S. humosa* DSM 5900^T^ [53]; 11, *T. pusilla* DSM 6293^T^ [56]; 12, *T. litoreum* CL-GR58^T^ [57]; 13, *T. lucentensis* QMT2^T^ [58]; 14, *Z. compransoris* LMG 5821^T^ [53]; +, Positive; −, negative; w, weak; nd, not determined. Summed feature 3, C_16 : 1_* ω*7*c* and/or C_16 : 1_* ω*6*c*; summed feature 8, C_18 : 1_* ω*7*c* and/or C_18 : 1_* ω*6*c*. Fatty acid data for *T. siberiense* 26-4b^T^ from this study.

Characteristic	1	2	3	4	5	6	7	8	9	10	11	12	13	14
Habitat	Oil sludge	Mesotrophic fen	Mud	Vinegar plant	Wheat	Biofilter	Marine macroalga	Freshwater	Salterns and salt lakes	Soil, freshwaters	Marine shellfish	Coastal seawater	Sea water	Mud
Gram reaction	Negative	Negative	Negative	Negative	Negative	Negative	Negative	Negative	Negative	Negative	nd	Negative	Negative	Negative
Morphology	Spiral	Vibrio, spirilla	Helical spirilla	Rod	Vibroid	Coccoid	Spiral	Rod	Vibrioid, spiral	Six-pronged stars	Spiral	Slightly curved rod	Vibrioid, spiral	Straight rod, curved rod
Motility	−	+	+	nd	nd	nd	+	−	+	−	+	+	+	+
Cells (μm)	0.8–3.0×0.4–0.6	Diameter 0.2–0.6	0.2–0.4×3–4	0.6–0.9×1.0–4.0	1.0–1.7	1.5–2.1	0.5–0.6×2.5–5.0	0.68–0.92×1.16–1.91	0.8–0.9×1.0–3.5	Diameter 0.7–3.0	0.3–0.5×1.2–4.0	0.3–0.5×1.3–1.5	3–5×0.6	0.7–0.9×0.5–2.1
Temperature range (optimum) for growth (°C)	20–30 (25)	4–30 (25-28)	18–38 (nd)	nd (30)	nd (37)	15–45 (30–35)	4–40 (25)	18–37 (30–35)	20–45 (42)	nd (28–30)	6–40 (32)	10–35 (30–35)	4–40 (nd)	nd
pH (optimum) for growth	7.0 (6.0–7.5)	4–7 (5.7–6.0)	1.5–1.5 (nd)	nd (4–6)	5.7–6.8 (nd)	5.5–11.0(8)	3.5–9.5 (3)	nd	nd (7.5–8.0)	Neutral	6.0–9.0 (nd)	7–9 (8)	nd	nd
NaCl range (optimum) for growth (%,w/v)	0 (nd)	nd	nd	nd	nd	nd	0.3–10.0 (nd)	nd	3–24 (8–12)	Up to 1	0.5–8.0 (nd)	1–10 (nd)	2–10 (nd)	nd
DNA G+C content (mol%)*	68.07	61.5–64.0	67.2	56.2–57.2	69–70	60.2–60.4	51.1	nd	69.1	69.3–72.9	48/51	68	54.7	66.1
API 20NE assay:														
Arginine	+	nd	nd	nd	nd	nd	nd	nd	nd	nd	nd	nd	nd	nd
Reduction of nitrate	−	−	nd	−	+ (to nitrite)	+	+ (to N_2_O)	+	nd	nd	+ (to nitrite)	+ (to nitrite)	nd	nd
Urease	+	nd	nd	nd	nd	+	−	w	nd	nd	nd	nd	nd	nd
Gelatin hydrolysis	+	nd	−	nd	nd	nd	nd	+	nd	nd	−	nd	−	−
Oxidase	−	−	+	−	+	+	+	+	nd	+	+	+	+	nd
Catalase	−	−	+	+	nd	+	+	−	nd	+	−	+	+	nd
Fatty acid	Summed feature 8, summed feature 3, iso-C_15 : 0_ 3-OH, iso-C_17 : 0_ 3-OH	C_18 : 1_* ω*7*c*, C_16 : 0_, C_17 : 0_	Summed feature 8, summed feature 3, C_16 : 0_	nd	nd	nd	C_18 : 1_* ω*7*c*, C_16 : 1_* ω*7*c*, C_16 : 0_, C_18 : 0_, C_19 : 0_ cyclo *ω*8*c*	nd	C_18 : 1_, C_18 : 0_	nd	C_14 : 0_ 3-OH, C_18 : 1_, C_16 : 1_, C_16 : 0_	C_16 : 0_, C_18 : 1_* ω*7*c*, C_17 : 0_	C_14_, C_16 : 0_ 3-OH, C_16 : 1_* ω*7*c*	C_18 : 1_, C_16 : 0_, C_18 : 0_ 3-OH, C_16 : 0_ 3-OH
Quinone	Q9, Q10	nd	Q10	Q-9	nd	nd	nd	nd	Q-10, MK-10	nd	Q-10	Q-10	nd	Q-10

Comparative phenotypic characteristics between strain Y-M2^T^ and the closely related type strain *T. siberiense* 26-4b^T^ are presented in Table 1. The representative species of the order *Rhodospirillales* are mesophilic, and contain Q-9 and/or Q-10 as the predominant quinones, with the exception of *Rhodovibrio salinarum* DSM 915^T^, which contains MK-10. However, there were many differences that distinguish strain Y-M2^T^ from its closely related species *T. siberiense* 26-4b^T^, as well as the other families within *Rhodospirillales*, such as gelatinase activity and pyruvate utilization (Table 1). The cellular fatty acid compositions of Y-M2^T^ and *T. siberiense* 26-4b^T^ were different (Table 1). Furthermore, a physiological comparison revealed that strain Y-M2^T^ had differences in terms of sulphur and nitrate metabolism from the representative species of the families ‘*Magnetospirillaceae*’, ‘*Novispirillaceae*’, and *Rhodospirillaceae* (Table 2). Meanwhile, strain Y-M2^T^ could be distinguished in terms of urease from ‘*Magnetospirillaceae*’, and by cell shape from most representative species in *Rhodospirillaceae*. Therefore, on the basis of phylogenetic analyses, lower genomic indices, and physiological traits, we propose that strain Y-M2^T^ represents a novel family in the order *Rhodospirillales*.

**Table 2.** Differential phenotypic characteristics of strain Y-M2^T^ compared with phylogenetically closed families within the order *Rhodospirillales* Taxa: 1, *Oleispirillaceae* fam. nov. (this study); 2, ‘*Magnetospirillaceae*’ [15,28, 31, 59, 60]; 3, ‘*Novispirillaceae*’ [10,61,63]; 4, *Rhodospirillaceae* [60,64,75]. +, Positive; −, negative; +/−, positive in some species but negative in others; w, weak; nd, not determined.

Characteristics	*Oleispirillaceae*	*Rhodospirillaceae*	*‘Novispirillaceae’*	*‘Magnetospirillaceae’*
Genera	1	14	5	5
Oxygen requirement	Microaerophilic	Strictly aerobic, aerobic or facultatively anaerobic	Anaerobic	Aerobic or microaerobic
Shape	Spiral	Rod, curved rod, vibrioid or spiral	Helical	Spirillum, vibrioid to spiral
Motility	+	+/−	+	+/−
Gram stain	−	−	−	−
Flagellation	Single polar	Single polar or bipolar tufts of flagella (two to five fibrils)	Single polar or bipolar	Polar or subpolar flagella
Oxidase	−	+/−	+/−	+/−
Catalase	−	+/−	+	+/−
Growth temperature (°C):				
Range	20–30	10–45	10–47	4–55
Optimum	25	25–40	25–37	25–45
NaCl concentration for growth (%):				
Range	0	0–15	0–12	0–3
Optimum	0	0–8	0.5–8.0	−
pH for growth:				
Range	7.0	5.0–11.0	6.5–10.0	4.0–10.0
Optimum	6.0–7.5	7.0–9.0	7.0–8.0	5.7–7.5
Gelatin hydrolysis	+	+/−	−	−
Urease	+	+/−	+/−	−
H_2_S production	nd	−	+/−	+
Electron acceptors (+)	nd	Sulphate and nitrate	Nitrate	Nitrate
Utilization of sugars	+	+	+	+
Utilization of amino acids	+	+	+	−
Utilization of organic acids	+	+	+	+
Respiratory quinones	Q9, Q10	Q-7, MK-7, Q8, RQ8, Q9, Q10	Q-9, Q-10	Q-9, MK-9, Q-10
Genome size (Mb)	3.2	2.1–5.4	2.4–4.6	3.8–6.2
DNA G+C content (mol%)	68.1	59.1–69.3	62.4–70.0	61.6–66.4

In conclusion, strain Y-M2^T^ belongs to the order *Rhodospirillales* and represents a novel genus in a novel family based on the results of phylogenetic analyses, genome relatedness, as well as the observed differences in physiological traits, for which the name *Oleispirillum naphthae* gen. nov. sp. nov. within *Oleispirillaceae* fam. nov. is proposed. The type strain is Y-M2^T^.

## Description of *Oleispirillum* gen. nov.

*Oleispirillum* (O.le.i.spi.ril’lum. L. neut. n. *oleum* oil; N.L. neut. dim. n. *spirillum*, a small spiral; N.L. neut. n. *Oleispirillum* small spiral associated with oil).

Gram-stain-negative, spiral, microaerophilic, mesophilic, motile, and non-spore-forming. The predominant cellular fatty acids are summed feature 3, iso-C_15  :  0_ 3-OH, summed feature 8, and iso-C_17  :  0_ 3-OH. Polar lipids include phosphatidylethanolamine, aminolipid and aminophospholipid. The predominant quinones are Q-9 and Q-10. The type species is *Oleispirillaceae naphthae*.

## Description of *Oleispirillum naphthae* sp. nov

*Oleispirillaceae naphthae* (naph’thae. L. gen. fem. n. *naphthae*, of oil).

Cells are Gram-stain-negative, spiral, motile with flagella, and non-spore-forming. Cell size is 0.8–3.0×0.2–0.4 µm. Growth is observed at 20–30 °C (optimal temperature 25 °C), pH 7.0 (optimal pH 6.0–7.5) without NaCl under 2%–10 % oxygen. Positive reactions for alkaline phosphatase, esterase (C4), lipid esterase (C8), leucine arylaminase, acid phosphatase, naphthol AS-BI-phosphate hydrolase, β-galactosidase, and β-glucosidase. Negative reactions for lipoid enzyme (C14), valine arylaminase, cystine arylaminase, trypsin, chymotrypsin, α-galactosidase, uronic acid glycosidase, α-glucosidase, *N*-acetylglucosamine enzyme, α-mannosidase, and β-fucosidase in API ZYM tests. Positive for acetoin (acetyl methylcarbinol) produced by fermentation of glucose by bacteria utilizing the butylene glycol pathway and the production of the enzyme gelatinase which liquefies gelatin in API 20E tests. Positive for arginine dihydrolase, urease, hydrolysis aesculin and β-galactosidase, negative for reduction of nitrates to nitrites or nitrogen in API 20NE tests. The predominant quinones are Q-9 and Q-10. The predominant cellular fatty acids are summed feature 3, iso-C_15 : 0_ 3-OH, summed feature 8 and iso-C_17  :  0_ 3-OH. Polar lipids mainly include phosphatidylethanolamine, aminolipid, and aminophospholipid.

The type strain, Y-M2^T^ (=CCAM 827^T^=JCM 34765^T^), was isolated from oil sludge. The G+C content of the genomic DNA of strain Y-M2^T^ is 68.1 mol%.

The almost complete 16S rRNA gene sequence and draft genome of strain Y-M2^T^ was deposited to GenBank under the accession MZ270535 and JAQAZG000000000, respectively.

## Description of *Oleispirillaceae* fam. nov

*Oleispirillaceae* (O.le.i.spi.ril.la.ce’ae. N.L. neut. n. *Oleispirillum* a bacterial genus; *-aceae* suffix to denote a family; N.L. fem. pl. n. *Oleispirillaceae* the *Oleispirillum* family).

The description of the family is based on the type genus *Oleispirillum*. This family is affiliated with the order *Rhodospirillales* in the class *Alphaproteobacteria*.

The type and only genus is *Oleispirillum*.

## supplementary material

10.1099/ijsem.0.006292 Uncited Supplementary Material 1.
