# Supplementary material for: Oleispirillum naphthae gen. nov., sp. nov., a bacterium isolated from oil sludge, and proposal of Oleispirillaceae fam. nov
Source: Int J Syst Evol Microbiol. 2024 Mar 21;74(3):006292. doi: 10.1099/ijsem.0.006292 (PMC10963914; doi:10.1099/ijsem.0.006292)
Supplement: Uncited Supplementary Material 1. [file ijsem-74-06292-s001.pdf]

Supplementary Materials

Table S1 Summary of the draft genome assembly of the Y-M2^T strain.

Draft genome feature	Length/Number
Genome Size / bp	3214613
ORF No.	3016
rRNA No. (5S, 16S, 23S)	3
tRNA No.	48
Contigs No.	27
G+C content	68.07
N20	461460
N50	216590
N90	128336
Hit in KofamKOALA	1970

Table S2 16S rRNA gene sequence similarities, average nucleotide identity (ANI), average amino acid identity (AAI), and percentage of conserved proteins (POCP) between strain Y-M2^T and representative species of family "*Magnetospirillaceae*", "*Novispirillaceae*", and *Rhodospirillaceae* clustered in the same phylogenetic clade. 16S rRNA gene sequence similarities were calculated based on phylogenetic analysis using pairwise distance. Bold number indicates the highest value of each index.

strain	family	species	Type strain	Accession number	16S rRNA similarities	ANI	AAI	POCP
Y-M2	"*Magnetospirillaceae*"	*Telmatospirillum siberiense*	26-4b1	GCA_027864655.1	**90.8**	70.00	**60.20**	41.08
Y-M2	"*Magnetospirillaceae*"	*Magnetospirillum moscoviense*	BB-1	GCA_001650635.1	90.3	67.05	58.18	42.08
Y-M2	"*Magnetospirillaceae*"	*Magnetospirillum kuznetsovii*	LBB-42	GCA_003284725.1	90.4	70.32	58.00	41.28
Y-M2	"*Magnetospirillaceae*"	*Magnetospirillum molischianum*	DSM 120	GCA_000294655.1	89.4	70.37	58.34	42.88
Y-M2	"*Magnetospirillaceae*"	*Magnetospirillum fulvum*	SMG 113	GCA_000442515.1	88.8	70.55	58.42	45.96
Y-M2	"*Magnetospirillaceae*"	*Magnetospirillum marisnigri*	SP 1	GCA_001650715.1	89.5	70.53	58.24	41.12
Y-M2	"*Magnetospirillaceae*"	*Magnetospirillum magneticum*	AMB-1	GCA_000009985.1	89.5	70.58	57.75	42.31
Y-M2	"*Magnetospirillaceae*"	*Magnetospirillum aberrantis*	SpK	GCA_011022235.1	89.6	70.28	58.26	45.30
Y-M2	"*Magnetospirillaceae*"	*Magnetospirillum caucaseum*	SO-1	GCA_000342045.1	89.4	71.10	57.97	40.17
Y-M2	"*Magnetospirillaceae*"	*Magnetospirillum gryphiswaldense*	MSR-1	GCA_002995535.1	90.6	70.36	58.06	43.09
Y-M2	"*Magnetospirillaceae*"	*Magnetospirillum magnetotacticum*	MS-1	GCA_000829825.1	89.5	69.95	57.70	40.48
Y-M2	"*Magnetospirillaceae*"	*Roseospira marina*	CE2105	GCA_008630015.1	89.8	71.02	58.00	43.67
Y-M2	"*Magnetospirillaceae*"	*Roseospira navarrensis*	SE 3104	GCA_009601025.1	88.8	70.97	58.08	42.89
Y-M2	"*Magnetospirillaceae*"	*Roseospira visakhapatnamensis*	JA131	GCA_014197915.1	87.4	71.62	58.34	43.11
Y-M2	"*Magnetospirillaceae*"	*Roseospira goensis*	JA135	GCA_014197795.1	89.3	69.45	58.46	44.10
Y-M2	*Rhodospirillaceae*	*Pararhodospirillum oryzae*	JA318	GCA_007992075.1	88.7	70.13	57.99	47.73

Y-M2	*Rhodospirillaceae*	*Rhodospirillum rubrum*	S1	GCA_000013085.1	89.3	70.38	58.37	47.26
Y-M2	*Rhodospirillaceae*	*Roseospirillum parvum*	930I	GCA_900100455.1	90.6	71.31	58.21	**48.03**
Y-M2	*Rhodospirillaceae*	*Phaeovibrio sulfidiphilus*	JA480	GCA_014861485.1	88.5	70.20	57.24	41.95
Y-M2	*Rhodospirillaceae*	*Rhodospirillum photometricum*	132	GCA_000284415.2	89.3	69.85	58.15	45.94
Y-M2	*"Novispirillaceae"*	*Haematospirillum jordaniae*	H5569	GCA_001611975.1	89.9	71.69	57.40	43.84
Y-M2	*"Novispirillaceae"*	*Caenispirillum salinarum*	AK4	GCA_000315795.1	89.7	70.89	57.97	42.22
Y-M2	*"Novispirillaceae"*	*Caenispirillum bisanense*	K92	GCA_900230255.1	90.2	**71.83**	58.65	43.07
Y-M2	*"Novispirillaceae"*	*Insolitispirillum peregrinum*	NCIB 9435	GCA_900156605.1	89.5	69.85	57.79	44.73
Y-M2	*"Novispirillaceae"*	*Novispirillum itersonii*	NCIB 9070	GCA_000381985.1	89.4	70.10	57.91	45.33

(A)

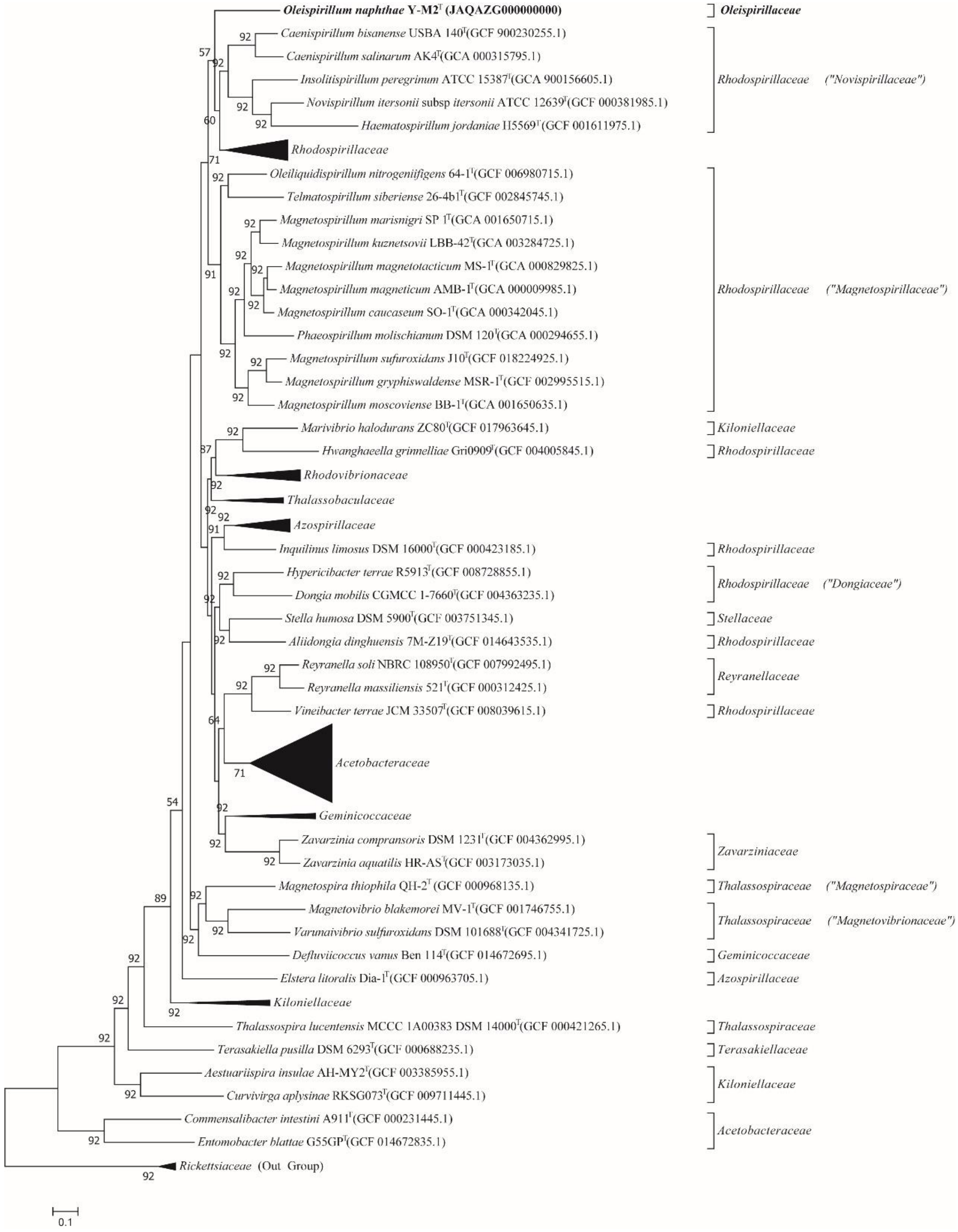
Oleispirillum naphthae Y-M2T (JAQAZG000000000)
Oleispirillaceae
Caenispirillum bisanense USBA 140T(GCF 900230255.1)
Caenispirillum salinarum AK4T(GCA 000315795.1)
Insolitispirillum peregrinum ATCC 15387T(GCA 900156605.1)
Novispirillum itersonii subsp itersonii ATCC 12639T(GCF 000381985.1)
Haematospirillum jordaniae H5569T(GCF 001611975.1)
Rhodospirillaceae ("Novispirillaceae")
Rhodospirillaceae
Oleiliquidispirillum nitrogeniifigens 64-1T(GCF 006980715.1)
Telmatospirillum siberiense 26-4b1T(GCF 002845745.1)
Magnetospirillum marisnigri SP 1T(GCA 001650715.1)
Magnetospirillum kuznetsovii LBB-42T(GCA 003284725.1)
Magnetospirillum magnetotacticum MS-1T(GCA 000829825.1)
Magnetospirillum magneticum AMB-1T(GCA 000009985.1)
Magnetospirillum caucaseum SO-1T(GCA 000342045.1)
Phaeospirillum molischianum DSM 120T(GCA 000294655.1)
Magnetospirillum sufuroxidans J10T(GCF 018224925.1)
Magnetospirillum gryphiswaldense MSR-1T(GCF 002995515.1)
Magnetospirillum moscoviense BB-1T(GCA 001650635.1)
Rhodospirillaceae ("Magnetospirillaceae")
Marivibrio halodurans ZC80T(GCF 017963645.1)
Kiloniellaceae
Hwanghaeella grinnelliae Gri0909T(GCF 004005845.1)
Rhodospirillaceae
Rhodovibrionaceae
Thalassobaculaceae
Azospirillaceae
Inquilinus limosus DSM 16000T(GCF 000423185.1)
Rhodospirillaceae
Hypericibacter terrae R5913T(GCF 008728855.1)
Dongia mobilis CGMCC 1-7660T(GCF 004363235.1)
Rhodospirillaceae ("Dongiaceae")
Stella humosa DSM 5900T(GCF 003751345.1)
Stellaceae
Aliidongia dinghuensis 7M-Z19T(GCF 014643535.1)
Rhodospirillaceae
Reyranella soli NBRC 108950T(GCF 007992495.1)
Reyranella massiliensis 521T(GCF 000312425.1)
Reyranellaceae
Vineibacter terrae JCM 33507T(GCF 008039615.1)
Rhodospirillaceae
Acetobacteraceae
Geminicoccaceae
Zavarzinia compransoris DSM 1231T(GCF 004362995.1)
Zavarzinia aquatilis HR-AST(GCF 003173035.1)
Zavarziniaceae
Magnetospira thiophila QH-2T (GCF 000968135.1)
Thalassospiraceae ("Magnetospiraceae")
Magnetovibrio blakemorei MV-1T(GCF 001746755.1)
Varunaivibrio sulfuroxidans DSM 101688T(GCF 004341725.1)
Thalassospiraceae ("Magnetovibrionaceae")
Defluviicoccus vanus Ben 114T(GCF 014672695.1)
Geminicoccaceae
Elstera litoralis Dia-1T(GCF 000963705.1)
Azospirillaceae
Kiloniellaceae
Thalassospira lucentensis MCCC 1A00383 DSM 14000T(GCF 000421265.1)
Thalassospiraceae
Terasakiella pusilla DSM 6293T(GCF 000688235.1)
Terasakiellaceae
Aestuariispira insulae AH-MY2T(GCF 003385955.1)
Curvivirga aplysinae RKSG073T(GCF 009711445.1)
Kiloniellaceae
Commensalibacter intestini A911T(GCF 000231445.1)
Entomobacter blattae G55GPT(GCF 014672835.1)
Acetobacteraceae
Rickettsiaceae (Out Group)
0.1

(B)

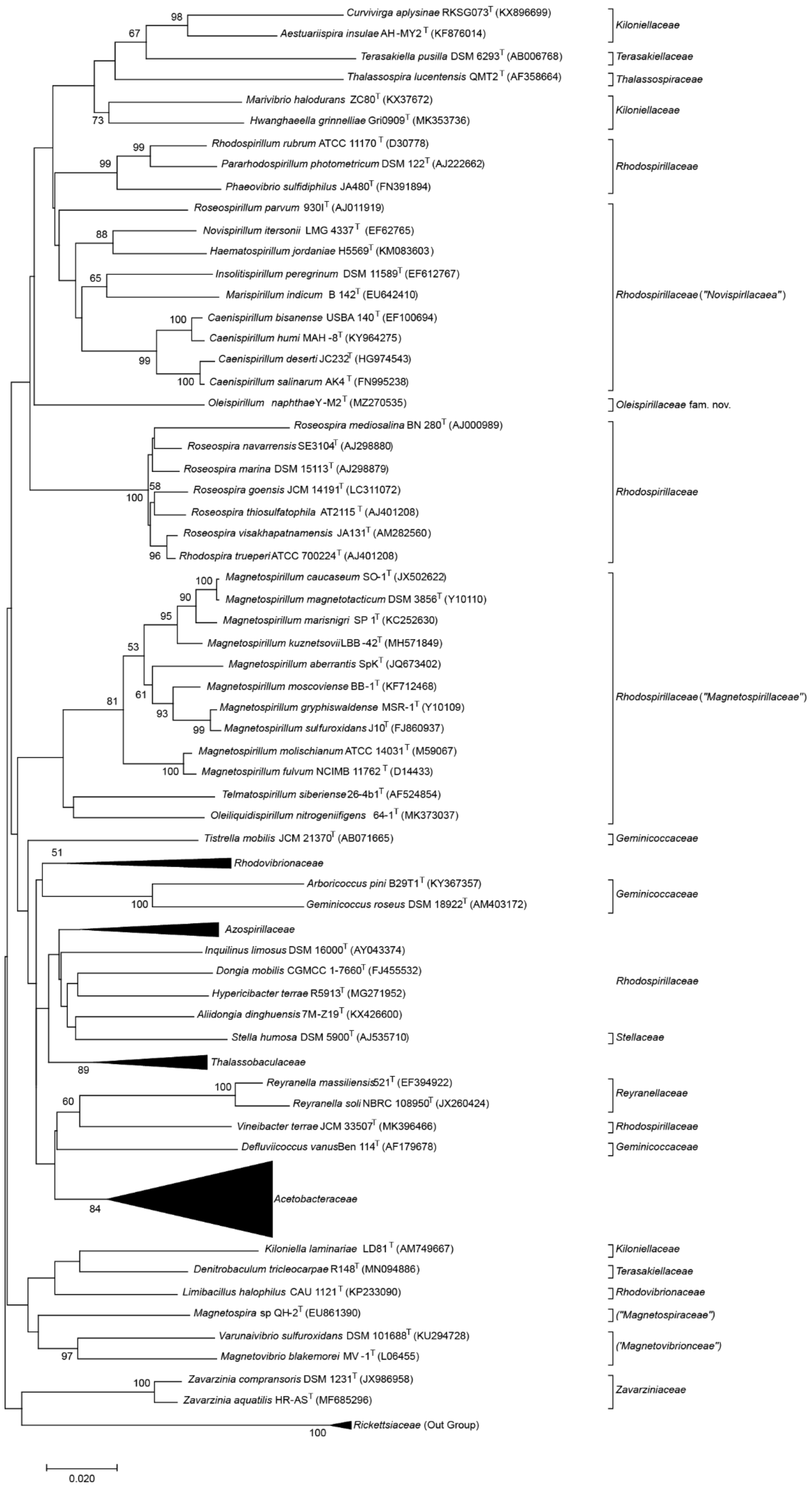

Curvivirga aplysinae RKSG073T (KX896699)
Aestuariispira insulae AH-MY2 T (KF876014)
Terasakiella pusilla DSM 6293T (AB006768)
Thalassospira lucentensis QMT2 T (AF358664)
Marivibrio halodurans ZC80T (KX37672)
Hwanghaeella grinnelliae Gri0909T (MK353736)
Rhodospirillum rubrum ATCC 11170 T (D30778)
Pararhodospirillum photometricum DSM 122T (AJ222662)
Phaeovibrio sulfidiphilus JA480T (FN391894)
Roseospirillum parvum 930IT (AJ011919)
Novispirillum itersonii LMG 4337 T (EF62765)
Haematospirillum jordaniae H5569T (KM083603)
Insolitispirillum peregrinum DSM 11589T (EF612767)
Marispirillum indicum B 142T (EU642410)
Caenispirillum bisanense USBA 140 T (EF100694)
Caenispirillum humi MAH -8T (KY964275)
Caenispirillum deserti JC232T (HG974543)
Caenispirillum salinarum AK4 T (FN995238)
Oleispirillum naphthae Y-M2T (MZ270535)
Roseospira mediosalina BN 280T (AJ000989)
Roseospira navarrensis SE3104T (AJ298880)
Roseospira marina DSM 15113T (AJ298879)
Roseospira goensis JCM 14191T (LC311072)
Roseospira thiosulfatophila AT2115 T (AJ401208)
Roseospira visakhapatnamensis JA131T (AM282560)
Rhodospira trueperi ATCC 700224T (AJ401208)
Magnetospirillum caucaseum SO-1T (JX502622)
Magnetospirillum magnetotacticum DSM 3856T (Y10110)
Magnetospirillum marisnigri SP 1T (KC252630)
Magnetospirillum kuznetsovii LBB -42T (MH571849)
Magnetospirillum aberrantis SpKT (JQ673402)
Magnetospirillum moscoviense BB-1T (KF712468)
Magnetospirillum gryphiswaldense MSR-1T (Y10109)
Magnetospirillum sulfuroxidans J10T (FJ860937)
Magnetospirillum molischianum ATCC 14031T (M59067)
Magnetospirillum fulvum NCIMB 11762 T (D14433)
Telmatospirillum siberiense 26-4b1T (AF524854)
Oleiliquidispirillum nitrogeniifigens 64-1T (MK373037)
Tistrella mobilis JCM 21370T (AB071665)
Rhodovibrionaceae
Arboricoccus pini B29T1T (KY367357)
Geminicoccus roseus DSM 18922T (AM403172)
Azospirillaceae
Inquilinus limosus DSM 16000T (AY043374)
Dongia mobilis CGMCC 1-7660T (FJ455532)
Hypericibacter terrae R5913T (MG271952)
Aliidongia dinghuensis 7M-Z19T (KX426600)
Stella humosa DSM 5900T (AJ535710)
Thalassobaculaceae
Reyranella massiliensis 521T (EF394922)
Reyranella soli NBRC 108950T (JX260424)
Vineibacter terrae JCM 33507T (MK396466)
Defluviicoccus vanus Ben 114T (AF179678)
Acetobacteraceae
Kiloniella laminariae LD81 T (AM749667)
Denitrobaculum tricleocarpae R148T (MN094886)
Limibacillus halophilus CAU 1121 T (KP233090)
Magnetospira sp QH-2T (EU861390)
Varunaivibrio sulfuroxidans DSM 101688T (KU294728)
Magnetovibrio blakemorei MV -1T (L06455)
Zavarzinia compransoris DSM 1231T (JX986958)
Zavarzinia aquatilis HR-AST (MF685296)
Rickettsiaceae (Out Group)
98
67
73
99
99
88
65
100
99
100
58
100
96
100
90
95
53
61
93
99
81
100
51
100
89
100
60
84
97
100
100
Kiloniellaceae
Terasakiellaceae
Thalassospiraceae
Kiloniellaceae
Rhodospirillaceae
Rhodospirillaceae ("Novispirllacaea")
Oleispirillaceae fam. nov.
Rhodospirillaceae
Rhodospirillaceae ("Magnetospirillaceae")
Geminicoccaceae
Geminicoccaceae
Rhodospirillaceae
Stellaceae
Reyranellaceae
Rhodospirillaceae
Geminicoccaceae
Kiloniellaceae
Terasakiellaceae
Rhodovibrionaceae
("Magnetospiraceae")
('Magnetovibrionceae")
Zavarziniaceae
0.020

(C)

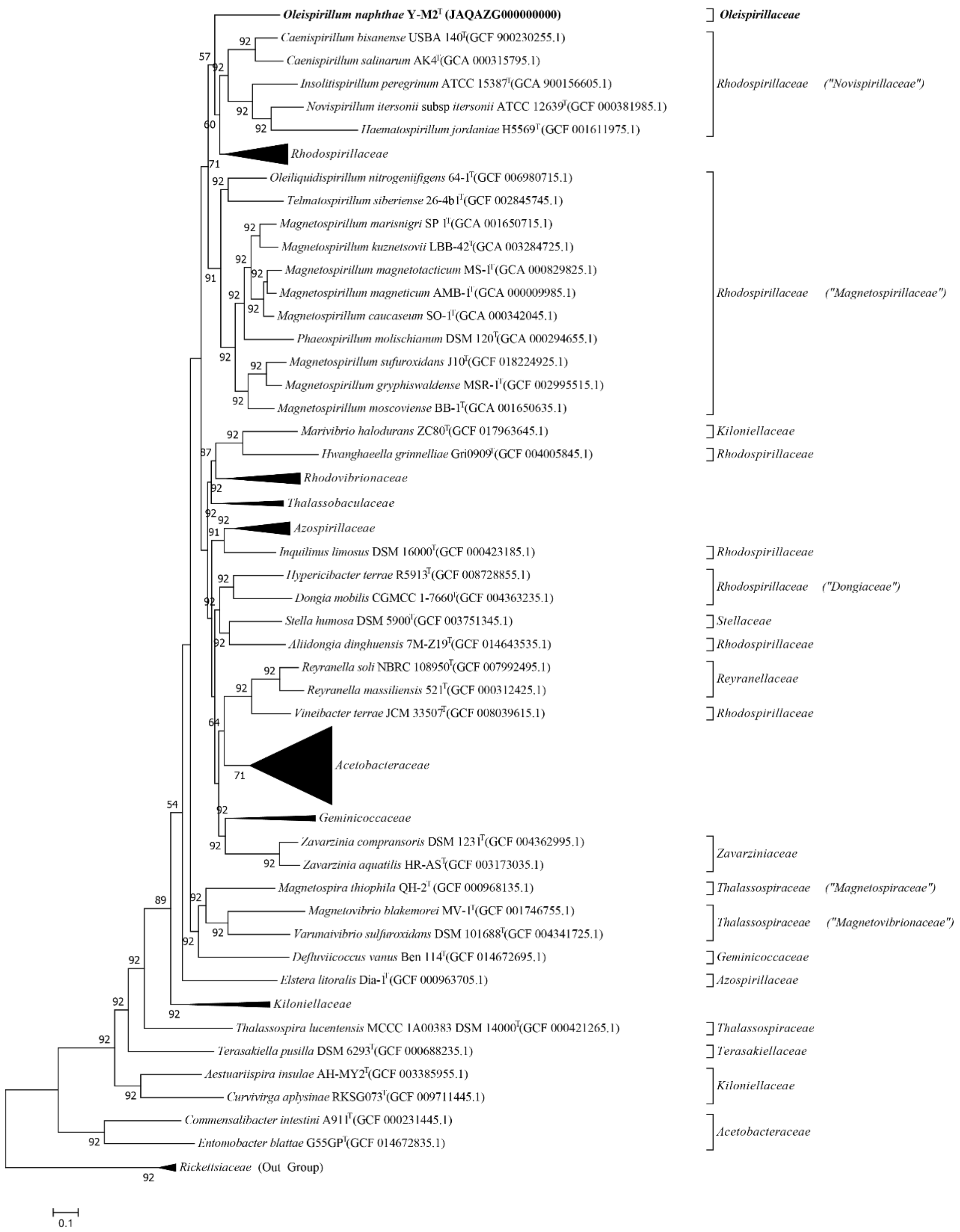

Fig S1. Phylogenetic trees of strain Y-M2^T and *Rhodospirilles* strains reconstructed based on 16S rRNA gene sequences using (A) neighbor-joining and (B) minimum-evolution algorithms, and (C) phylogenomic tree reconstructed using concatenated alignment of 92 core genes. Species of the genus *Rickettsia* within order *Rickettsiales* were used as an out-group. Bootstrap percentages are based on 1000 replications.

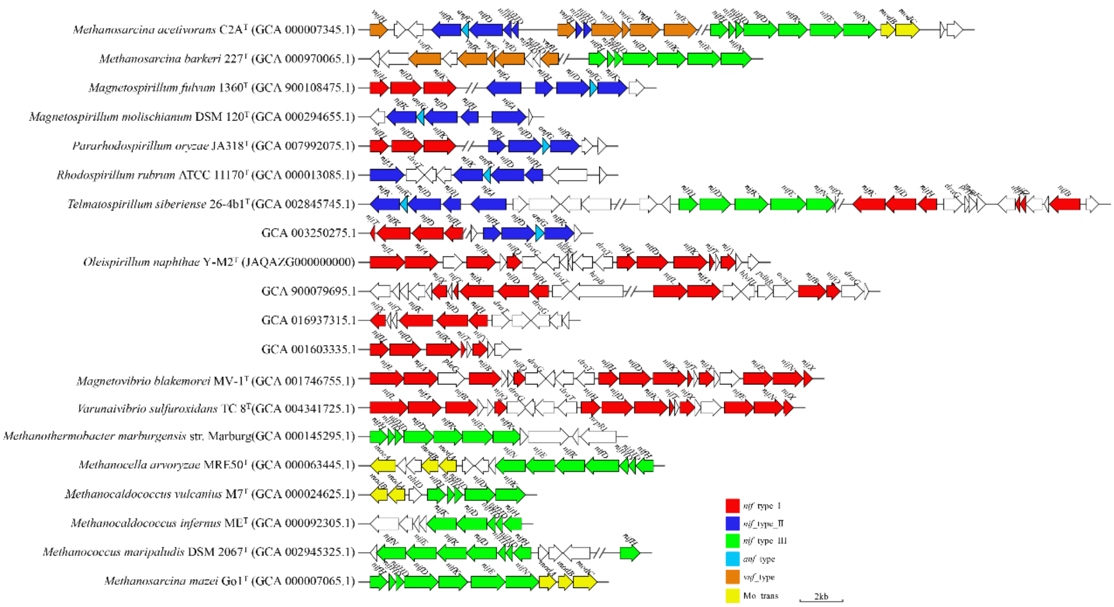

Fig S2. Organization of *nif*, *vnf*, *anf,* and *nif*-like genes in N_2-fixing strains of *Rhodospirillales* species and 8 archaea nitrogen-fixing species contain in lineage IV in fig S1. The three *nif* cluster types, *nif*_type_I, *nif*_type_II, and *nif*_type_III, are marked with red, blue and green, respectively. The *anf*_type and *vnf*_type genes were marked with light blue and brown, respectively. The gene relative to the Mo transform is marked with yellow.

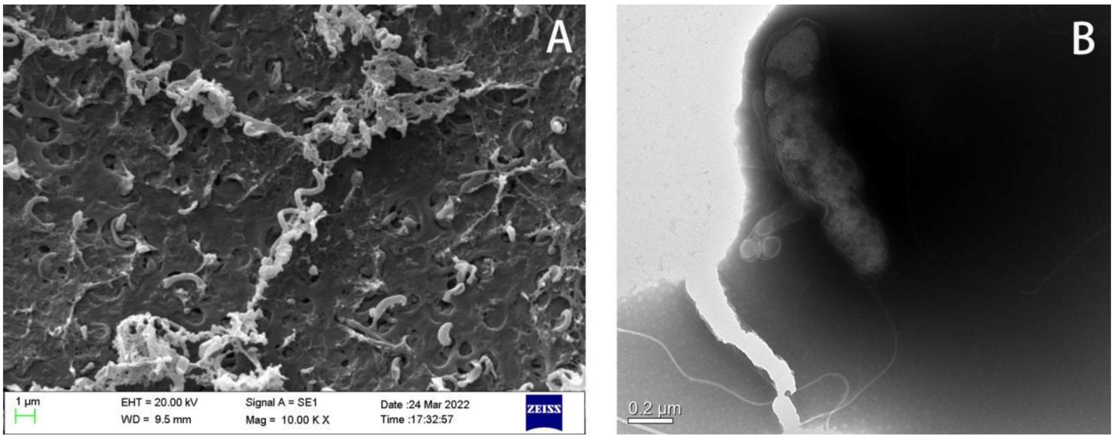

Fig S3. Micrographs of strain Y-M2^{T}. (A) Scanning electron micrograph. (B) Transmission electron micrograph.

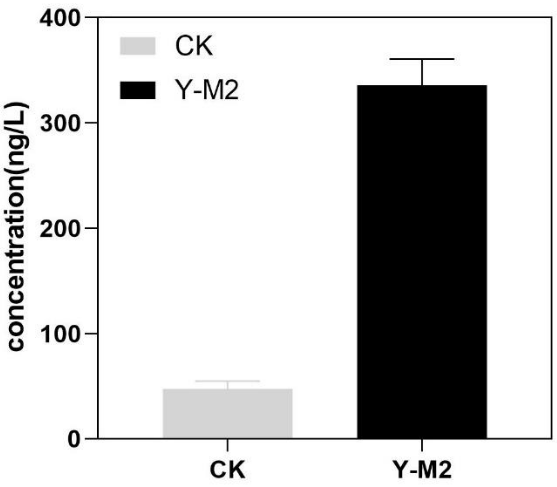

Fig S4. The nitrogenase concentration of strain Y-M2^{T} was determined by ELISA scientific research kit. CK, blank without culture; Y-M2, an extract obtained from broken cells of strain Y-M2T. The experiments were performed in triplicate.

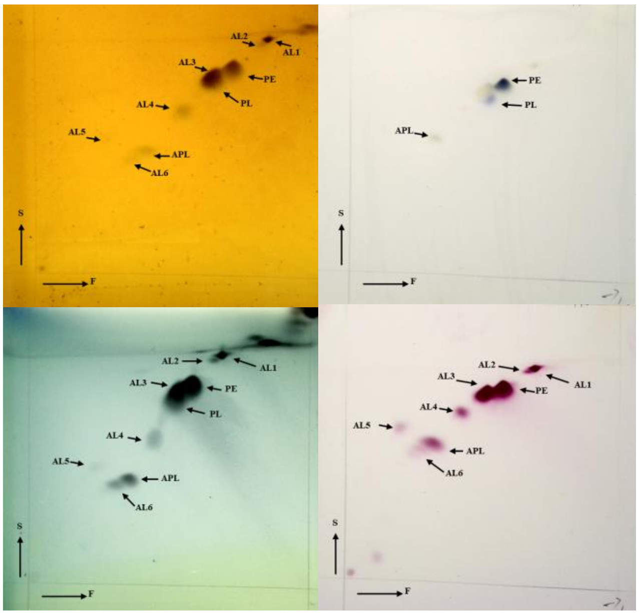

Fig S5. The major polar lipids of strain Y-M2T. (A) Staining with sulfuric alcohol; (B) Staining with phospholipid reagent;(C) Staining with phosphomolybdic acid; (D) Staining with ninhydrin;AL, aminolipid; APL, aminophospholipid; DPG, diphosphatidylglycerol; GL, glycolipid; L, unknown polar lipid; PC, phosphatidylcholine; PE, phosphatidylethanolamine; PG, phosphatidylglycerol; PME, phosphatidylmonomethylethanolamine; PL, phospholipid; SGL, sphingoglycolipid. F-first dimension of TLC; S- second dimension of TLC.
